# The Effects of an mRNA Covid-19 Vaccine Booster on Immune Responses in Cancer-Bearing Veterans

**DOI:** 10.18103/mra.v10i7.2932

**Published:** 2022-07-31

**Authors:** Arthur E. Frankel, Tazio Capozzola, Raiees Andrabi, Chul Ahn, Panpan Zhou, Wan-ting He, Dennis R. Burton

**Affiliations:** 1 Department of Medicine, West Palm Beach VA Medical Center, West Palm Beach, FL; 2 The Scripps Research Institute, La Jolla, CA, USA; 3 Division of Biostatistics, Department of Population and Data Sciences, University of Texas Southwestern Medical School, Dallas,TX, USA

**Keywords:** SARS-CoV-2, mRNA vaccines, antibodies, neutralizing antibodies, B cell malignancies

## Abstract

Immunocompromised cancer patients are at significant risk of severe acute respiratory syndrome coronavirus 2 (SARS-CoV-2) infection. A method to identify those patients at highest risk is needed so that prophylactic measures may be employed. Serum antibodies to SARS-CoV-2 spike protein are important markers of protection against COVID-19 disease. We evaluated total and neutralizing antibody levels pre and post third booster vaccine and compared responses among different cancer-bearing and healthy veterans. This as a prospective, single site, comparative cohort observational trial. The setting was the West Palm Beach VA Medical Center cancer center. All veterans received a third SARS-CoV-2 mRNA booster. The main outcomes were anti-SARS-CoV-2 spike IgG and neutralizing antibodies to wild-type, and B.1.617, BA1, BA2, and BA4/5 variants were measured. Disease type and therapy, COVID-19 infection, and anti-CD20 antibody treatments were documented. The third mRNA vaccine booster increased the mean blood anti-spike IgG five-fold. The second anti-spike level was equal or greater than the first in 129/140 veterans. All the groups except the myeloma group, had post-booster antibody levels significantly higher than pre-booster with 4-fold, 12-fold, 4-fold, 6-fold and 3.5-fold increases for the control, solid tumor, CLL, B cell lymphoma and all B cell malignancy cohorts. The myeloma set showed only a non-significant 1.7-fold increase. Recently anti-CD20 antibody-treated patients were shown to have approximately 200-fold less anti-S IgG production after vaccine booster than other patients. There was a 2.5-fold enhancement of wild-type virus mean neutralizing antibodies after a third mRNA booster and mean neutralization of Delta and Omicron variants increased 2.2, 6.5, 7.7, and 6.2-fold versus pre-boost levels. B cell malignancies failed to show increased post-booster neutralization. The third SARS CoV-2 booster increased total anti-spike IgG and neutralizing antibodies for most subjects. Veterans with B cell malignancies particularly myeloma and those receiving anti-CD20 monoclonal antibodies had the weakest humoral responses. Neutralizing antibody responses to Omicron variants were less than for wild-type virus. A subset of patients without humoral immunity post-booster should be considered for prophylactic antibody or close monitoring.

## Introduction

The persistence of the SARS-CoV-2 pandemic beyond three years with the emergence of novel virus variants poses particular health risks for immunocompromised cancer patients. While coronavirus disease 2019 (COVID-19) vaccine boosters in normal and non-hematologic malignancy patients yield excellent total and wild-type neutralizing antibody (nAb) increments,^1^ lower frequency and levels of response occur in B cell malignancy patients^2^—particularly in those treated with anti-CD20 monoclonal antibody or high dose corticosteroids.^3^ The Omicron variant of SARS-CoV-2 has 34 amino acid substitutions in receptor-binding domain residues that are frequently targeted by nAbs induced by the SARS-CoV-2 mRNA BNT162b2 and mRNA-1273 vaccines. Hence, vaccine-induced antibody reactivities are diminished for this variant.

Recent observational studies of total and nAbs generated by third vaccine boosters included relatively few subjects—27 solid tumor patients and 79 and 48 hematologic malignancy patients, respectively.^4–6^ Further, only one report correlated total anti-spike antibody concentrations with nAb titers.^6^ Thus, there is modest data to date. Nevertheless, findings of differences in total and neutralizing antibody titers are important as higher total and neutralizing antibody concentrations correlate with reduced COVID-19 hospitalizations and death.^7–10^ Based on this previous work and the large number of immunocompromised B cell malignancy veterans undergoing active therapy for progressive disease at VA hospitals, we conducted this prospective clinical investigation to quantify the anti-SARS CoV-2 spike antibodies pre and post third mRNA booster vaccines in controls, solid tumor and B cell malignancy (chronic lymphocytic leukemia, B cell lymphoma, and multiple myeloma) veterans at the West Palm Beach VA Medical Center between September, 2021 to April, 2022.

## Methods

We conducted a prospective, single site, observational cohort study in order to assess the humoral response to a third dose of mRNA vaccine (BNT162b2 or mRNA-1273) in 160 veterans with either no malignancies, advanced solid tumors, B cell lymphoma, multiple myeloma or chronic lymphocytic leukemia. All veterans were followed at the West Palm Beach VA Medical Center, West Palm Beach, FL. The primary endpoint was the increase of IgG anti-spike total and nAbs after the third booster. Secondary endpoints were correlation of total and neutralizing antibody titers and analyses of disease type, age, gender, race, prior anti-CD20 antibody exposure, and prior COVID-19 infection on antibody titers. The study was approved by the West Palm Beach VA Research and Education Committee and the Bay Pines Institutional Review Board (#1645747-1 approved September 7, 2021). The study was conducted in accordance with the declaration of Helsinki. All subjects signed written informed consent before enrollment. Vaccination history was confirmed, and data collected on age, gender, race, dates of vaccinations, prior COVID-19 infection, active and recent cancer therapies including anti-CD20 antibody infusions. Blood samples were obtained pre-third booster and one month post-booster, and sera isolated, aliquoted and stored at −80°C until assayed. Antibodies to SARS-CoV-2 wild-type spike protein were enumerated using the Abbott chemiluminescent microparticle immunoassays for IgG anti-spike as recommended by the manufacturer (see Supplemental Text).^11^ The limit of detection was 50AU/mL for each assay, and the upper limit of the assay was 50,000AU/mL. Neutralizing antibody to wild-type (WT), Delta variant (B.1.617.2), and Omicron variants (BA.1, BA.2, and BA.4/5) were assayed at The Scripps Research Institute. Under BSL2/3 conditions, MLV-gag/pol and MLV-CMV plasmids were co-transfected into HEK293T cells along with full-length or variously truncated SARS-CoV-1 and SARS-COV-2 spike plasmids using Lipofectamine 2000 to produce single-round of infection competent pseudoviruses. The medium was changed 16 h post transfection. The supernatant containing MLV-pseudotyped viral particles was collected 48 h post transfection, aliquoted and frozen at −80°C for the neutralization assay. Pseudotyped viral neutralization assays were performed as previously described with minor modification (see Supplemental Text).^12^ Dilution of serum yielding 50% inhibition of viral entry (ID50), or 50% neutralization titer (NT), was directly related to the quantity of neutralizing antibody. NT of ≤ 30 was undetectable neutralization. Data was described with the mean and standard deviation if continuous and as counts and percentage if categorical. Changes between pre-booster and post-booster were examined using a paired t-test. Comparisons between two groups were done using Student’s two-sided t-test or one-way ANOVA test with GraphPad Prism 9.3 software. A two-sided P value of <.05 was considered statistically significant.

## Results

Between September 13, 2021 and February 22, 2022, 160 veterans seen at the West Palm Beach VA cancer center who had received two prior doses of mRNA vaccine were recruited in this study. Twenty veterans either expired or withdrew consent prior to completing the study. Of the 140 remaining study subjects, the mean age was 74 with interquartile range (IQR) of 67–79 years (see Table 1). Most were male—137 out of 140 or 98%. Most were Caucasian—97 Caucasians (69%), 21 African American (15%), 18 Hispanic (13%), 2 Native American (1%), 1 Middle Eastern (1%) and 1 Pacific Islander (1%). The mean age of the full cohort is 74 years with an interquartile range of 68–79 years and overall range of 41–94 years. There were 55 solid tumor bearing veterans, 32 controls (non-solid tumor, non-B cell malignancies, and benign blood disorders), 19 B cell lymphoma patients, 22 B cell chronic lymphocytic leukemia (CLL), and 12 myeloma patients. 79 veterans had progressive disease on chemotherapy, and 61 had stable disease not on treatment. 15 veterans were treated with anti-CD20 monoclonal antibodies; two veterans had prior bone marrow transplants; and 14 subjects had COVID-19 of which 8 had infection prior to booster and 6 after booster (see Supplemental Text).

The third mRNA vaccine booster increased the blood anti-spike IgG from 5,903 ± 12,530 AU/mL to 30,362 ± 19,699 AU/mL. The post/pre antibody ratio was 5.1. The change was statistically significant with P<0.0001. The second anti-spike level was equal or greater than the first in 129/140 veterans. Of the remaining eleven veterans, six showed minimal changes in AU/mL and five showed larger changes. Four of these veterans had inter-assay intervals of 3, 3.5, 4 and 5 months whereas most of the samples were obtained 1–2 months apart. Thus, the peak immune response may have decayed by the second assay point. The fifth veteran had COVID-19 one month prior to the third booster, and that may have caused a transient antibody spike. A total of 17/140 subjects had both pre-booster and post-booster anti-spike antibody levels of <1,000 AU/mL. The maximum titer on these veterans were 0, 5, 7, 10, 13, 18, 24, 27, 44, 60, 61, 103, 136, 155, 427, 896 and 954 AU/mL. These patients included four with lymphoma on anti-CD20 antibody treatment, one with lymphoma on chemotherapy, four with CLL on treatment, three with myeloma on treatment and five additional patients without clear reason for poor seroconversion. These five veterans included a stable CLL, a 91 year old with no active malignancy, an 85 year old with prostate cancer, an 86 year old with metastatic melanoma on temozolomide, and a metastatic renal cell carcinoma on axitinib and pembrolizumab.

Analysis of the effects of disease on seroconversion was then addressed (Table 2 and Figure 2). All the groups except the myeloma group, had post booster antibody levels significantly higher than pre-booster with 4-fold, 12-fold, 4-fold, 6-fold and 3.5-fold increases with P values of <0.0001, <0.0001, 0.005, 0.0009 and <0.0001 for the control, solid tumor, CLL, B cell lymphoma and all B cell malignancy cohorts, respectively. In contrast, the myeloma set showed only a 1.7-fold increase that was not statistically significant. The set of all B cell malignancy veterans yielded lower post-booster anti-spike IgG levels than controls by 35% and lower than solid tumor veterans by 31% with P = 0.02 for both. Therapy effects on anti-spike IgG levels were scrutinized next (Table 2). Untreated, stable patients had a 30% better sero-response than treated cancer patients with P = 0.02. Among anti-CD20 antibody-treated patients treated within 21 months, the inhibition of anti-S IgG production after vaccine boost was over 200-fold when compared with patients treated with anti-CD20 antibody over 2 years prior to the study and for those never receiving anti-CD20 antibodies with P = 0.002 and P = 0.006, respectively.

**Figure 3.**
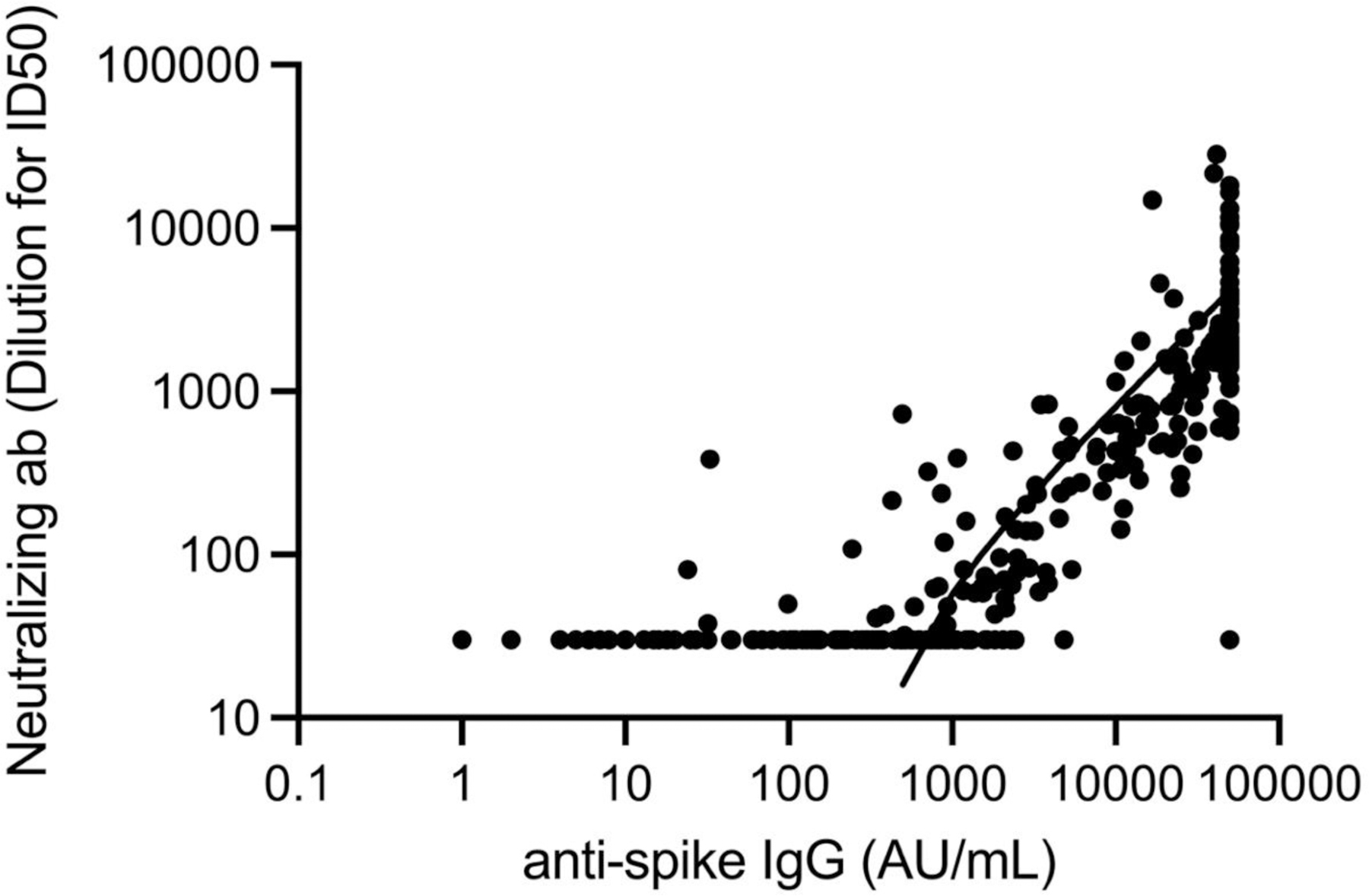
Plot of anti-S IgG AU/mL versus WT NT for all subjects. Linear regression analysis shows equation NT = 0.297 x anti-S IgG in AU/mL yields good fit with R^2^ = 0.26 and P=0.0036.

**Figure 4.**
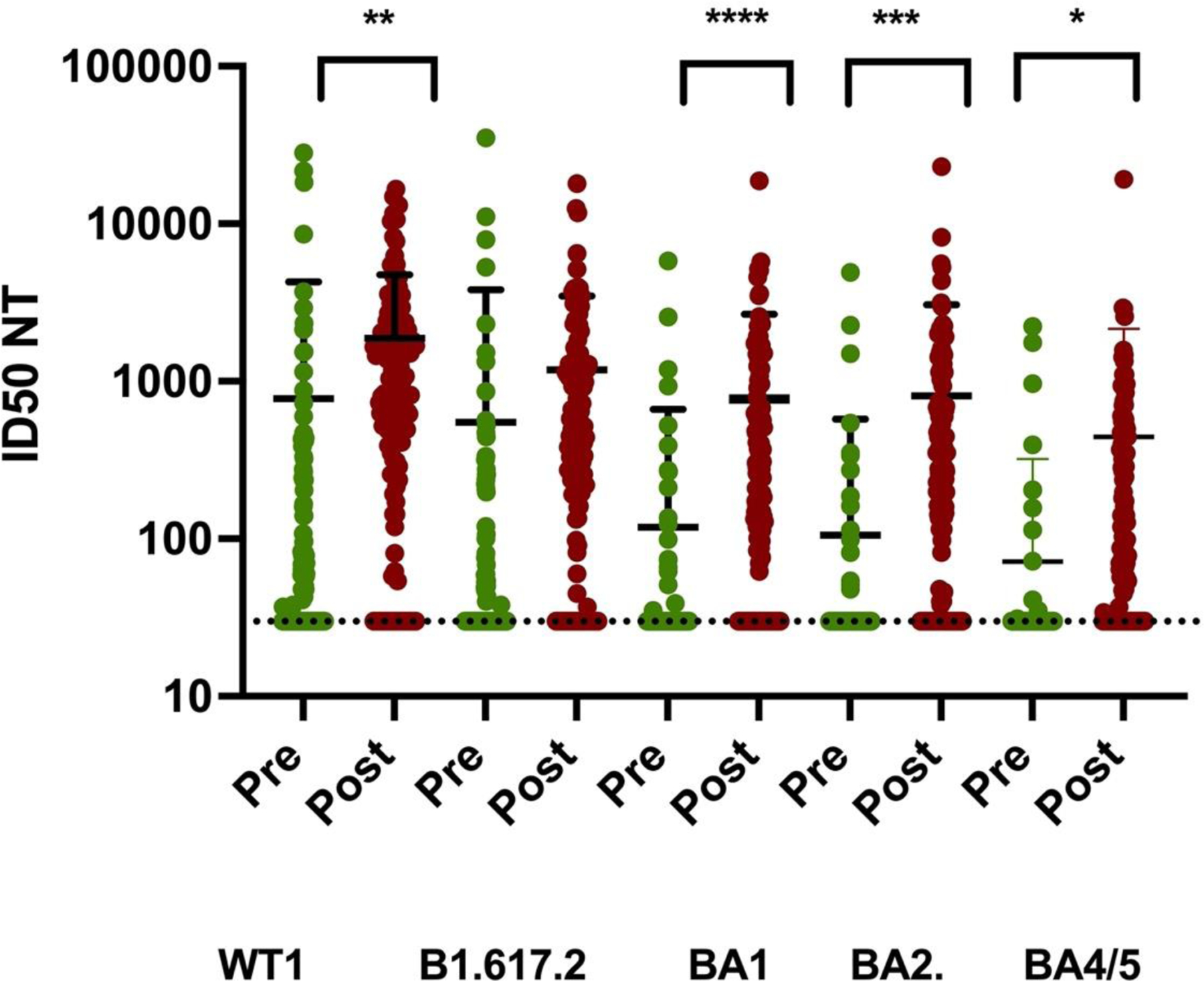
Pre and post third SARS-CoV-2 mRNA booster effect on pseudovirus Neutralizing Titer (NT) ID_50_. Statistical significance was 0.004 (**) for WT, 0.53 for B1.617.2, 0.0001 for BA1 (****), 0.0004 for BA2 (***), and 0.01(*) for BA4/5 for difference between pre-booster and post-booster NT. Mean and standard deviation shown on each column. Pre-boost samples in green, and post-boost samples in brown.

Virus neutralization was investigated next. For wild-type SARS-CoV-2 neutralization for the entire 140 enrolled veterans, there was a significant 2.5-fold enhancement in wild-type (WT) virus mean neutralization titer (NT) after a third mRNA booster (Table 3). When all of the chemiluminescent anti-spike IgG values were compared with matching viral WT NTs by linear regression analysis, the line described by NT = 0.297 x anti-spike IgG AU/mL gave an R^2^ of 0.26, F=95 and P<0.0001 for a statistically significant positive slope (Figure 3). Neutralization of variants was also improved by booster (Figure 4). Specifically, Delta, Omicron BA1, Omicron BA2, and Omicron BA4/5 NTs increased 2.2, 6.5, 7.7, and 6.2-fold versus pre-boost levels. While improved, the NTs were 61%, 41%, 43% and 24% of those against WT virus. These differences from WT neutralization were significant with P =.03, 0.0002, 0.0008, and 0.0001, respectively. The pre to post booster improvement in neutralization was not significant for B1.617.2 but significant for BA1, BA2, and BA4/5 with P values of 0.55, 0.0001, 0.0004, and 0.01, respectively. Only 1/140 subjects displayed a decrease in post-booster neutralization and that subject had a pre-booster wild-type neutralization of 64 and no neutralization of B1.617.2, BA1, BA2, or BA4/5. We next queried the number and characteristics of veterans with no viral neutralization by our assays. We found 15/140 subjects with no SARS-CoV-2 neutralization. These subjects had a median anti-spike IgG level of 291 ± 531 AU/mL versus positive SARS-CoV-2 neutralization subjects who had a median anti-spike IgG of 30,289 ± 18,686 AU/mL. This difference is significant with Student’s two-tailed t-test P<0.0001. Among these 15 patients without SARS-CoV-2 neutralization were four with B-cell lymphoma including three treated with anti-CD20 antibody, 3 with myeloma undergoing chemotherapy, and two with CLL on therapy. The remaining six included a CLL who recently stopped a BTK inhibitor, a myelodysplasia (MDS) on azacitidine, a cholangiocarcinoma on chemotherapy, an 85 year old with prostate cancer, an 86 year old with metastatic melanoma on temozolomide, and a metastatic renal cell carcinoma on axitinib and pembrolizumab. Many of these subjects matched with the low anti-spike IgG set described above. Because of the rarity of strong post-booster neutralizing responses, we closely examined the 5/140 with Omicron BA2 titers >4000. The highest responder paradoxically had a history of HIV-positive lymphoma, was on anti-retrovirals and had had prior anti-CD20 antibody years before. He had normal CD4 and CD8 T cell levels and no measurable circulating HIV RNA. The other four included a 69 year old female with CLL and a history of COVID-19 in December, 2020, a 71 year old male with cirrhosis, a 79 year old female with CLL on BTK inhibitor and prior anti-CD20 antibody x years before and a 64 year old male with mild renal insufficiency and anemia.

As a major goal of the study, we studied the effects of disease on post-booster NTs. The third booster produced a significant increase in NT for WT and all variants in control and solid tumor subjects (Table 3 and Table 4 and Figure 5A,B). There were no significant effects of chemotherapy on the solid tumor subjects’ post-booster NT increase or on the post-booster NT increase of all the patients even though total anti-spike IgG increment post booster was significantly reduced as noted above. In contrast, the B cell lymphoma, myeloma and CLL, and combined B-cell malignancy cohorts did not show significant post-booster changes in NT. The myeloma cohort post-NTs were less than those of control and solid tumor patients. Recent anti-CD20 therapy had a dramatic inhibitory effect on NT, but the small number (n = 4) of recently treated anti-CD20 subjects likely prevented statistical significance. Finally, we addressed the effects of age and race on anti-spike IgG and post NTs. The total and NTs were correlated with age in a linear regression analysis and both total anti-spike IgG and NTs to WT and BA2 did not show a correlation with R^2^’s of 0.01 to 0.03 and no strongly significant P values (see Figure 6A–C). For ethnicity, categories of Caucasian, African American and Hispanic versus post anti-spike IgG, post NT for WT and each variant were examined (Figure 7A,B). For the total post booster anti-spike IgG, there was no significant difference by ethnicity based on one-way ANOVA. Unexpectedly, African Americans had slightly greater post NT than Caucasians or Hispanics. The subgroups were too small to assess the effects of gender.

## Discussion

The evolution of the SARS-CoV-2 pandemic in the U.S. and the world necessitates a re-evaluation of prophylactic measures to protect the immunocompromised populations including veterans undergoing cancer therapy. With the emergence of Omicron variants containing 34 mutations in the spike protein and possessing immune evasion and enhanced transmissibility, the role of mRNA vaccine boosters is critical. A few studies have examined third mRNA vaccine boosters in cancer patients. Zeng described 27 solid tumor cancer patients with increased total and nAb titers post-booster to WT and variants including Omicron in agreement with our results.^5^ Fendler researched 199 cancer patients and found a third BNT162b2 mRNA booster yielded 90% and 56% detectable Omicron NT in solid tumor and hematologic cancers, respectively.^4^ Those receiving anti-CD20 monoclonal antibodies and most receiving BTK inhibitors failed to have detectable Omicron NT. We showed similar effects of recent anti-CD20 therapy. Bellusci evaluated 11 myelodysplasia and acute myeloid leukemia patients after a third mRNA booster.^6^ 4/11 had strong NT to WT (>1000) but weak against Omicron NT’s of 30, 52, 169 and 256); and 7/11 had weak NT to WT and none to Omicron. Their detectable range was >10. Interestingly, these investigators correlated Omicron NT with an anti-Omicron spike IgG enzyme-linked immunoassay (ELISA). The assay had an R = 0.66 and P = 0.0003 for correlation of ELISA and NT assay but the accuracy depended upon addition of 14 controls. They had similar success correlation of a WT ELISA with WT NT assay. Their work accords with our results on anti-S IgG and WT NT. Lasagna enrolled 142 cancer patients getting a third BNT162b2 mRNA booster and found almost all showed a brisk increase in total anti-spike IgG and WT NT with a correlation between the two assays (R = 0.27, P = 0.008).^1^ On ten of these cancer patients, Delta and Omicron variant NT assays were performed; there was a 12-fold decrease in Delta NT and a 32-fold decrease in Omicron NT.

Our results both confirm and extend the observations described. Both solid tumor and hematologic malignancy veterans in our study showed increases in total and neutralizing antibody with the third booster. But the immune responses were less in the B cell malignancies and particularly for the Omicron variants. Again, the correlation of total anti-spike IgG and NT fits with Bellusci and our present work. Multiple other studies reported a third mRNA booster in cancer patients but without Omicron information. Rottenberg tested 37 solid tumor cancer patients before and after a third BNT162b2 mRNA booster and all had increases in anti-spike IgG without a significant impact of chemotherapy.^13^ No NT was tested on WT or variants. We similarly did not see a major impact of treatment on booster responses. Shapiro gave third mRNA boosters to 88 cancer patients including 57 with hematologic cancers and observed decreased anti-spike IgG for hematologic cancer patients (particularly B cell malignancies and those receiving anti-CD20 antibody or BTK inhibitor.^14^ Again, our data matched with lower immune responses in our B cell malignancy cohorts. Debie gave third BNT162b2 mRNA vaccines to 141 hematologic malignancy participants.^15^ Again, anti-spike IgG rose except in anti-CD20 treated patients. Interestingly, their 30% rate of protocol dropout pairs with our 13% dropout rate and suggests compliance issues with COVID-19 vaccine boosters. Mair and Berger administered third BNT162b2mRNA vaccines to 439 cancer patients and 41 control health workers.^16^ Only anti-spike IgG was measured and there was no work on variants or neutralization assays. They validated the increase in anti-spike IgG and the reduced response to hematologic malignancy patients and found a higher anti-spike IgG increment in normal health care workers. Their most interesting observation was that ant-spike IgG correlated with peripheral blood CD19+ B and CD56+ NK cells.

The clinical relevance of our work is dependent on four findings. First, can we identify cancer patients that lack adequate total and neutralizing antibody to ameliorate the course of SARS-CoV-2 variant infections? Second, do patient’s humoral antibody responses predict serious infections? Third, can you employ prophylactic measures or diligent monitoring and early therapeutic interventions to protect high-risk patients? Fourth, how does the evolving landscape of variants impact our efforts? Our work suggests we can address the first question with available clinical tools. The anti-spike IgG assay is available at most hospitals in the U.S. We defined 17/140 veterans with <1,000 AU/mL post-third booster. These same veterans had no NT for WT or variants except for one patient with 954 AU/mL anti-spike IgG and who had WT NT of 119. The variant NTs for this patient were background—30. Thus, a potential group of high-risk cancer immunosuppressed patients were uncovered. There are clinical studies that establish low anti-spike IgG or absent virus neutralization is associated with more frequent infections of greater severity.^17–20^ Feng correlated anti-spike IgG and WT NT with ChAdOx1CoV-19 adenovirus vaccine protection from symptomatic B1.1.7 disease and found 80% protection was associated with 40,900 AU/mL and pseudovirus NT of 185 with P’s of 0.003 and 0.005, respectively.^17^ Anti-S IgG and WT NT correlated as well with R=0.66. Gilbert calculated WT pseudotyped NT as a marker for mRNA-1273 vaccine efficacy from any SARS-CoV-2 infection.^18^ A continuum model of COVID-19 risk decrement was found with increased WT NT with NTs of 10, 100 and 1000 associated with 78%, 91% and 96% disease prevention. Again, this work was done during prevalence of WT and Alpha B1.1.7 virus and application to B1.716.2 and Omicron variants is unknown. Thus, we have the tools to identify high risk patients. The vaccine booster non-responders may be offered prophylactic tixagevimab and cilgavimab monoclonal antibody treatments (Evusheld). The Evusheld cocktail is active against Omicron BA1 and BA2.^21^ Alternatively, close monitoring of high risk patients with home rapid antigen tests can be used to detect early infection and permit intervention. A number of the home rapid antigen tests are sensitive to Delta and Omicron infection.^22^ Once positive, high-risk patients may be treated with Paxlovid (nirmatrelvir and ritonavir). Paxlovid is active on Omicron variants.^23^ These temporizing measures may mitigate some of the dangers for immunocompromised cancer veterans who do not mount humoral immunity to vaccines. Finally, the recent emergence of Omicron BA.4 and BA.5 variants with immune escape from current vaccines and prior SARS-CoV-2 infections was reported in 54 patients (27 vaccinated and 27 recent infections) in New England.^24^ NTs were reduced many-fold. Their data matches our findings with BA.4/5 and suggests new approaches such as re-engineered vaccines may be needed. As noted above, in the interim, prophylactic antibody and early treatment anti-viral drugs may still reduce disease severity. The next few years will test our commitment to both viral surveillance, prophylaxis and intervention.

## Supplementary Material

supplementary material

## Figures and Tables

**Figure 1.**
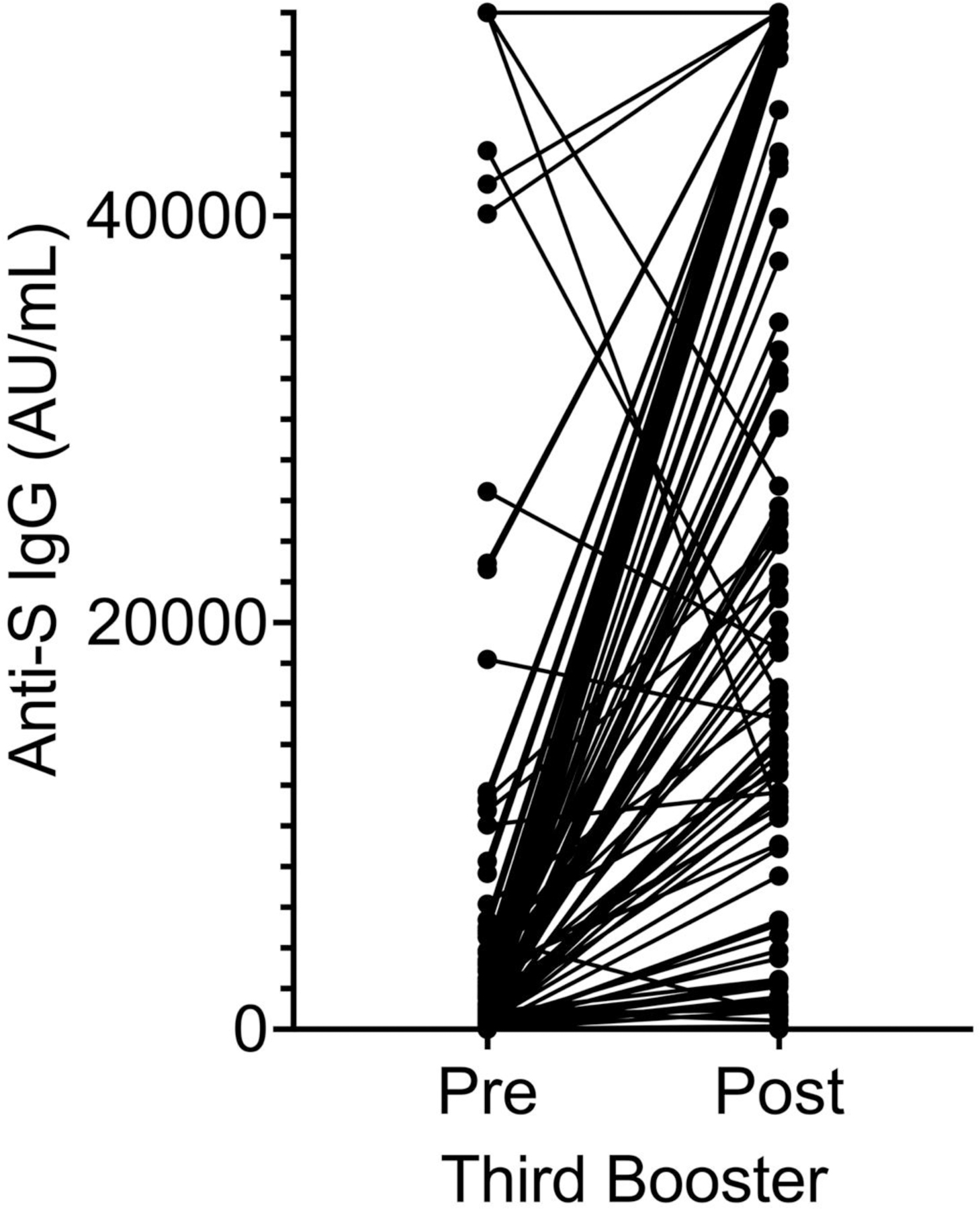
Anti-spike IgG circulating concentrations pre and post third booster. The second anti-spike antibody level was equal or greater than the first in 129/140 veterans. Of the remaining eleven veterans, six showed minimal changes in AU/mL(25 to 18, 16 to 13, 2 to 0, 8 to 7, 69 to 60, 933 to 477 AU/mL) and five showed larger changes (50,000 to 10,745, 18,190 to 15,300, 43,213 to 16,404, 26,452 to 18,702, 50,000 to 26,718 AU/mL). Four of these veterans had inter-assay intervals of 3, 3.5, 4 and 5 months whereas most of the samples were obtained 1–2 months apart. Thus, the peak immune response may have decayed by the second assay point. The fifth veteran had COVID-19 one month prior to the third booster, and that may have caused a transient antibody spike.

**Figure 2A.**
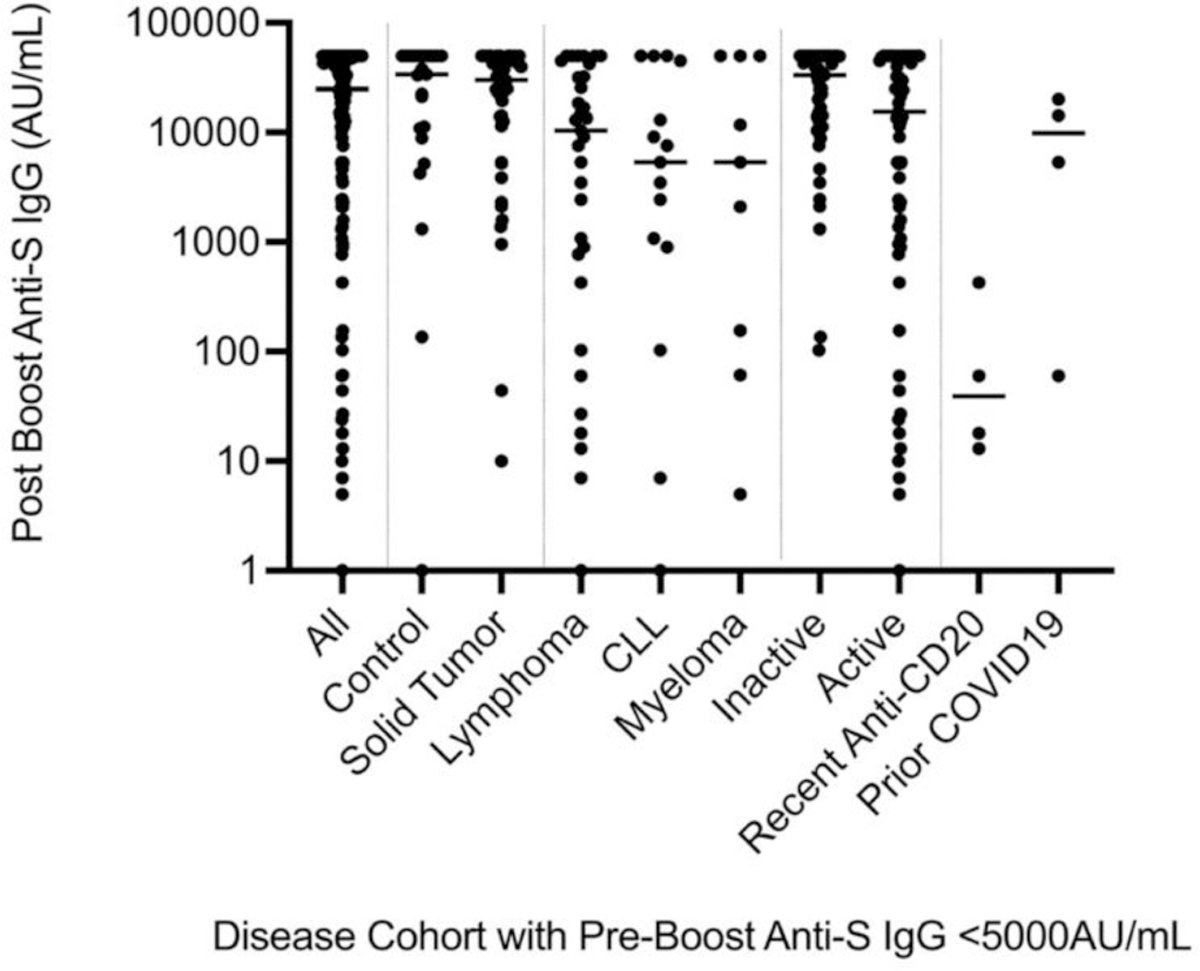
Chemiluminescence Assay. Anti-SARS-CoV-2 spike antigen IgG for each veteran with pre-booster levels <5,000AU/mL. Only Anti-CD20 antibody treated patients shown treated within 21 months of study. Among veterans developing COVID-19, only veterans getting illness before the second booster and with pre-booster level <5,000AU/mL are shown under COVID-19 column. This requirement excluded two veterans who developed COVID-19 after the booster and second blood collection and nine veterans with high pre-booster anti-S IgG levels. Thus, only 5 COVID-19 veterans displayed.

**Figure 2B.**
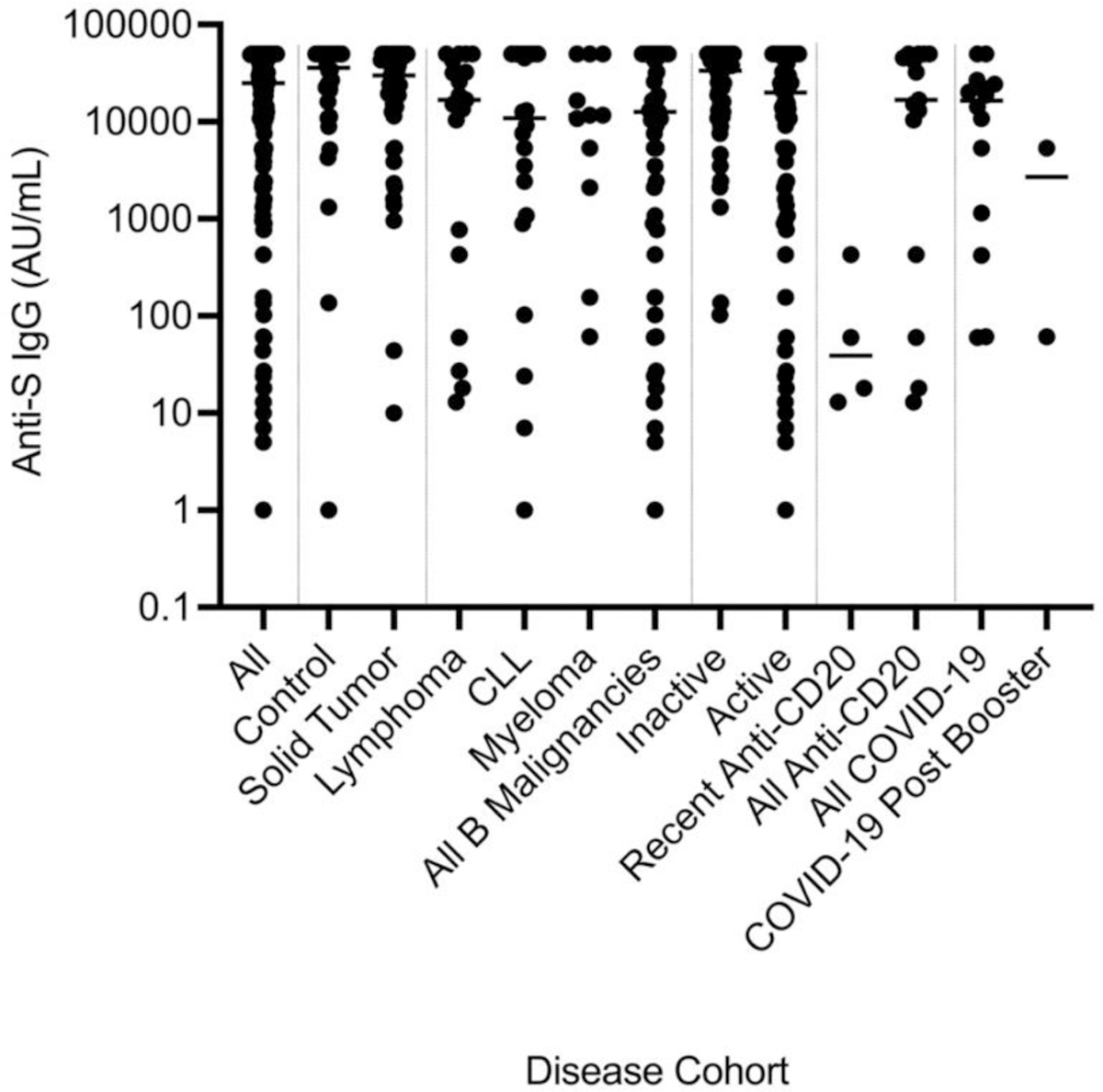
Chemiluminescence Assay. Anti-SARS-CoV-2 spike antigen IgG for each veteran independent of pre-booster anti-spike IgG titer.

**Figure 5A.**
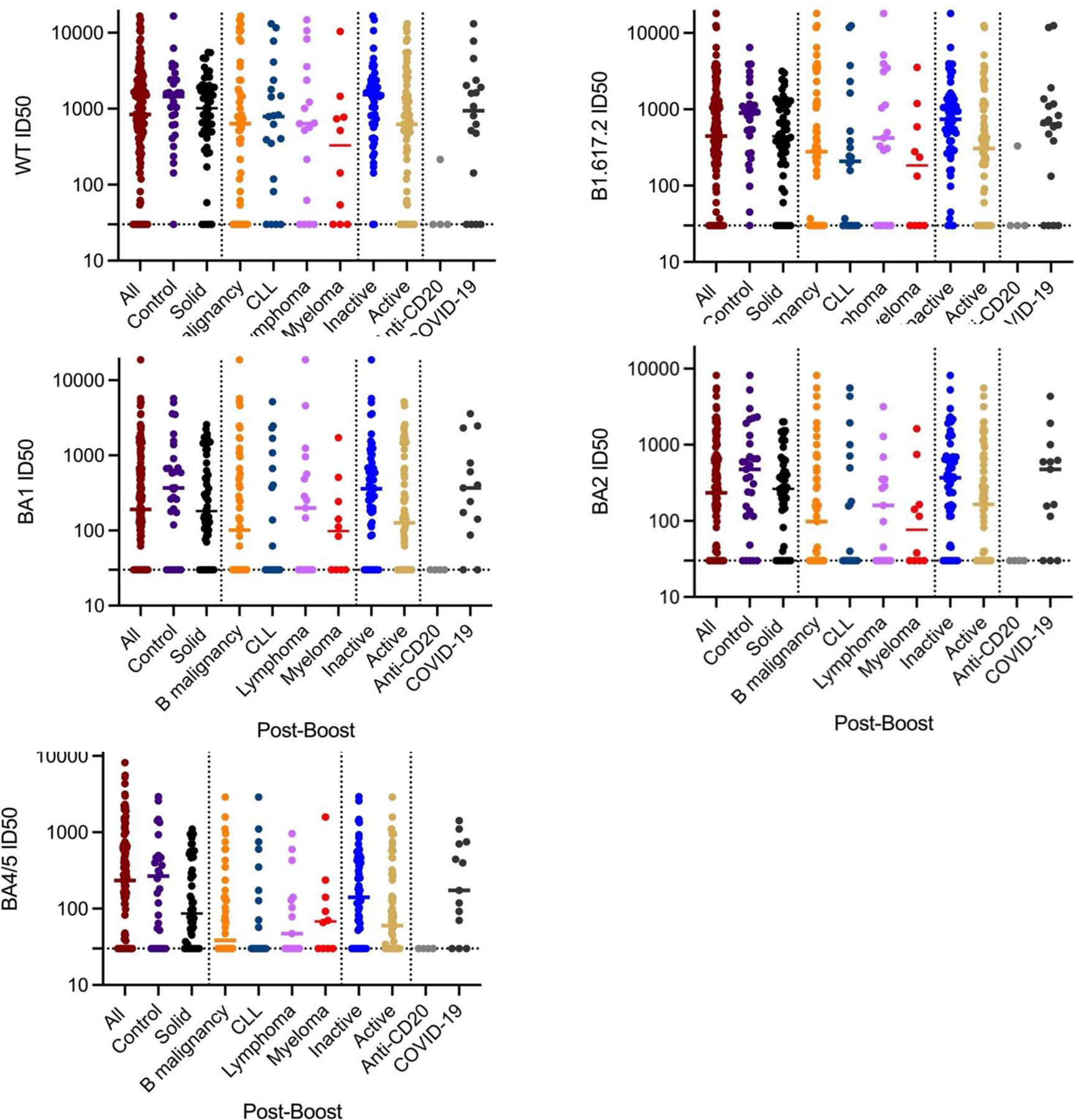
Post-third booster NT for disease cohorts. Anti-CD20 are only patients who received antibody within 21 months of booster. COVID-19 patients are all veterans who developed COVID-19 prior to or after booster. Controls are non-B cell heme malignancies and benign hematology. Solid refers to solid tumor patients. Lymphoma are all B-cell lymphomas. Inactive is untreated and stable; active is treated and progressive.

**Figure 5B.**
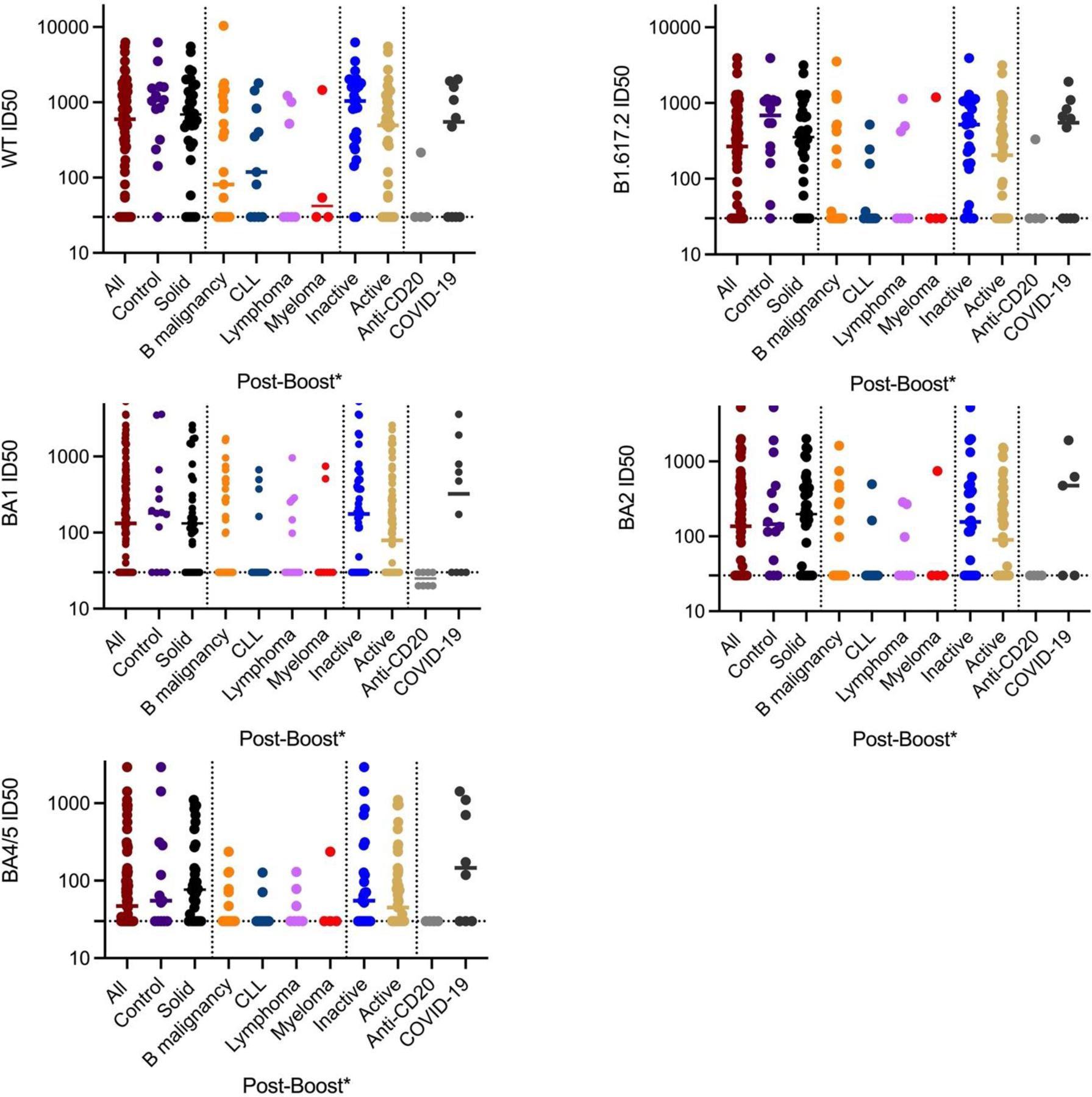
Post-third booster NT for disease cohorts for patients with ≤30 serum ID50 pre-boost(*). Anti-CD20 are only patients who received antibody within 21 months of booster. COVID-19 patients are all veterans who developed COVID-19 prior to or after booster. Controls are non-B cell heme malignancies and benign hematology. Solid refers to solid tumor patients. Lymphoma are all B-cell lymphomas. Inactive is untreated and stable; active is treated and progressive.

**Figure 6A.**
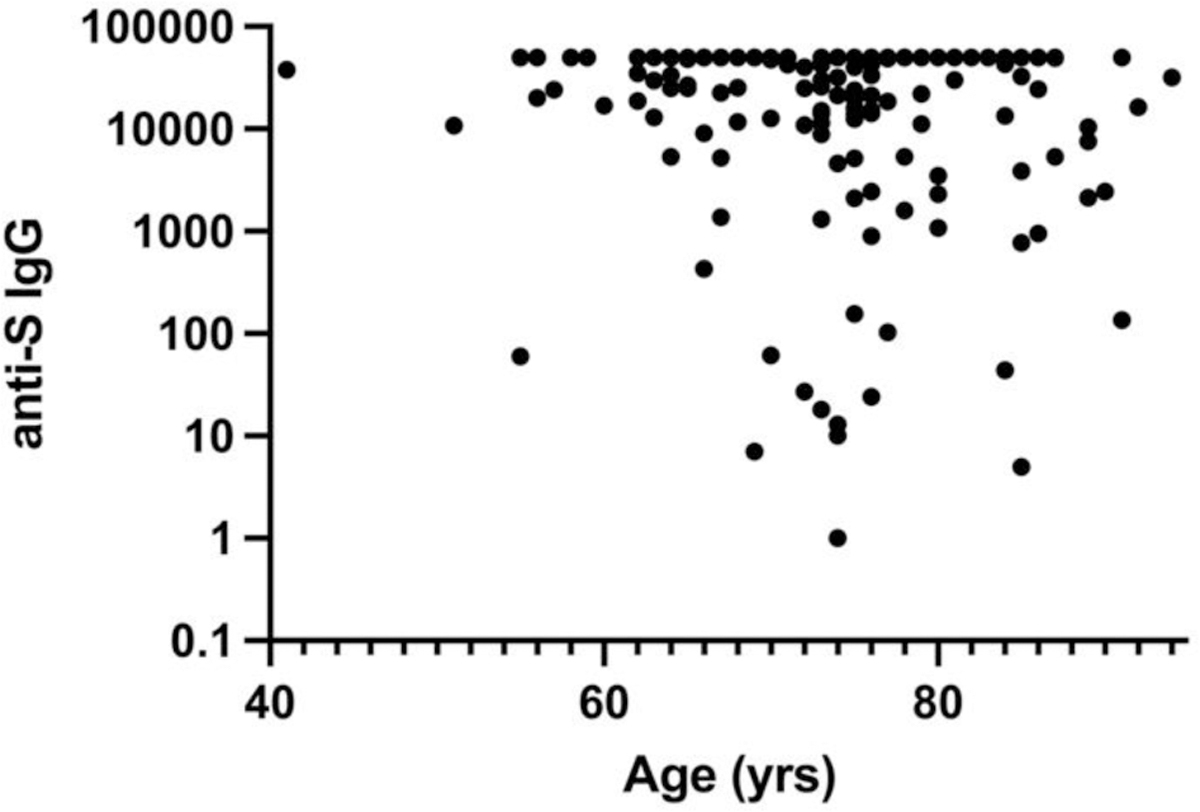
Effect of Age on post-booster anti-SARS CoV-2 spike IgG. Linear regression fit showed no significant correlation with R^2^ = 0.01, F = 2.46 and P = 0.12.

**Figure 6B.**
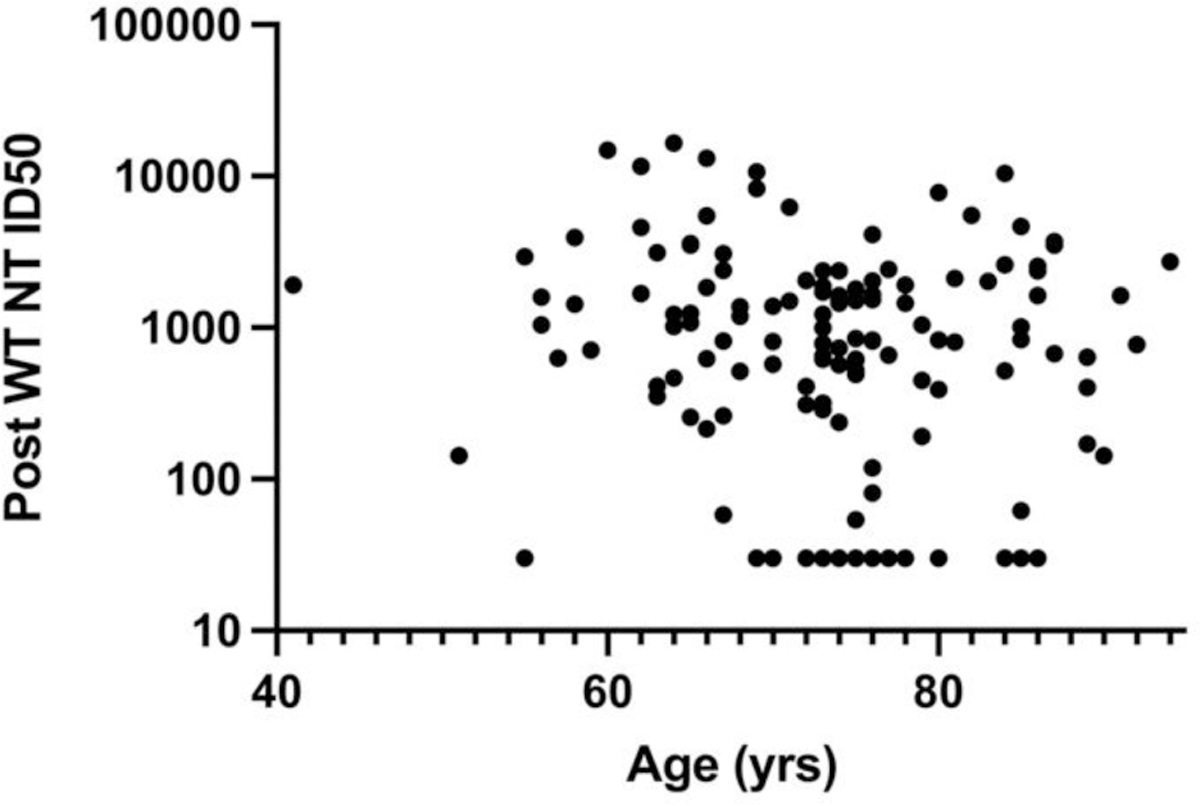
Effect of Age on post-booster WT NT. Linear regression fit showed no significant correlation with R^2^ = 0.02, F = 3 and P = 0.08.

**Figure 6C.**
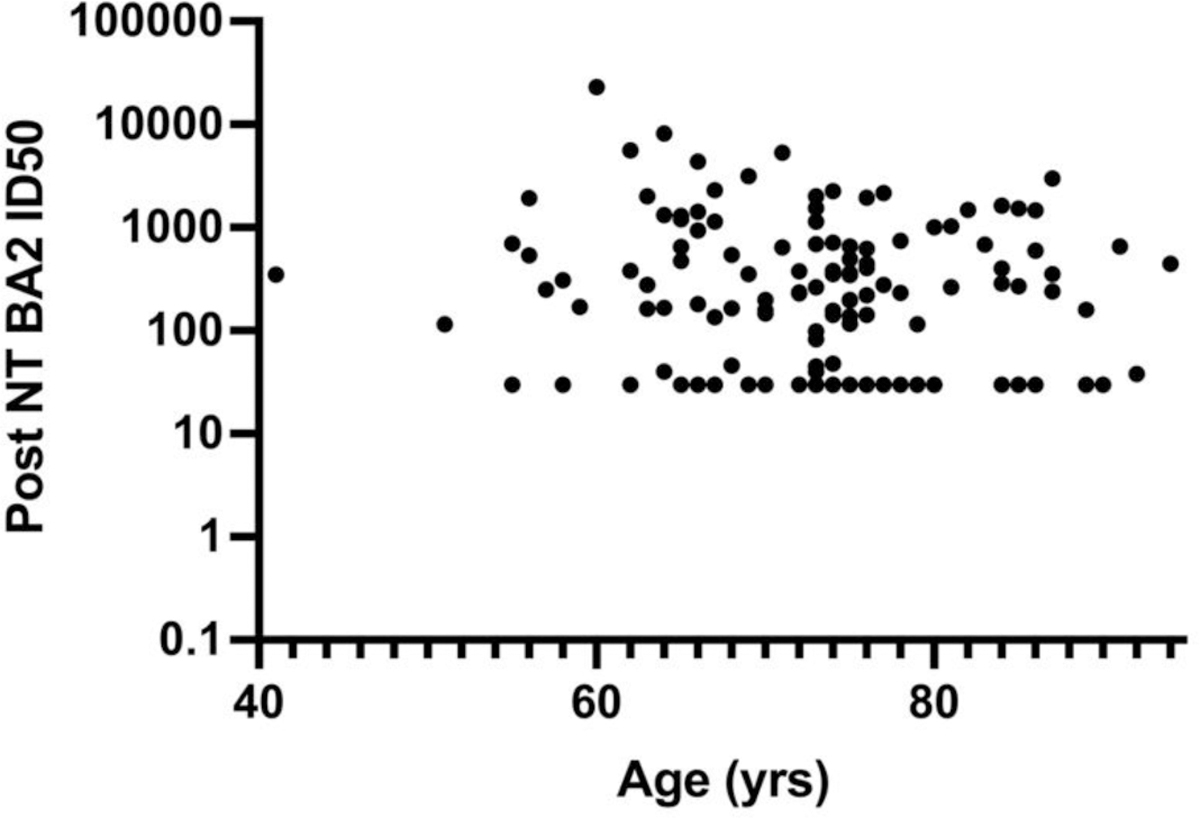
Effect of Age on Post-booster BA2 NT. Linear regression fit showed significant correlation with R^2^ = 0.03, F = 4 and P = 0.04 to equation BA2 NT = − 43 x Age + 39.

**Figure 7A.**
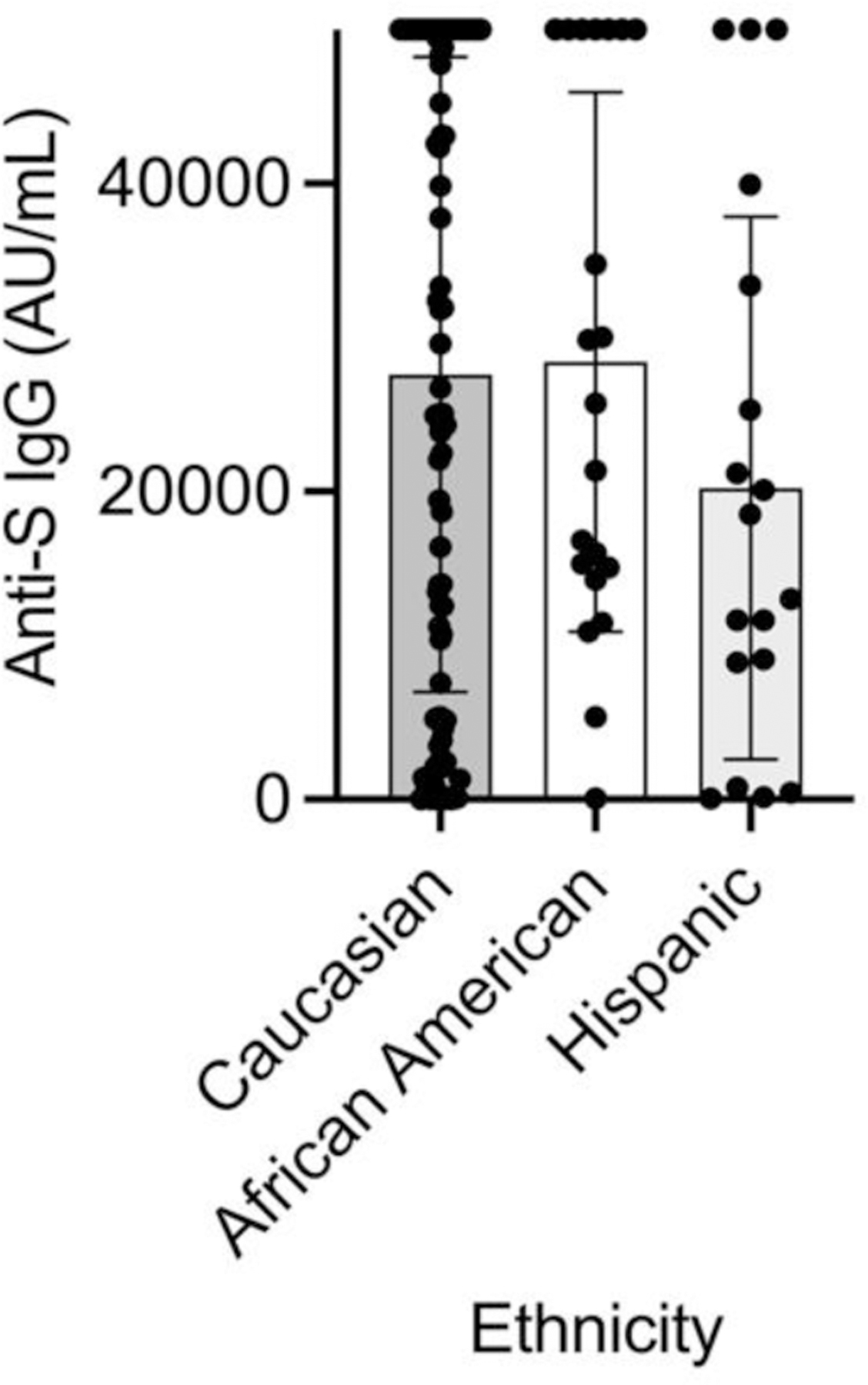
Effect of ethnicity on post-booster anti-spike IgG. For the total post-booster anti-spike IgG, there was no significant difference by race based on ordinary one-way ANOVA with F=1.1, R^2^=.01, and P=0.32.

**Figure 7B.**
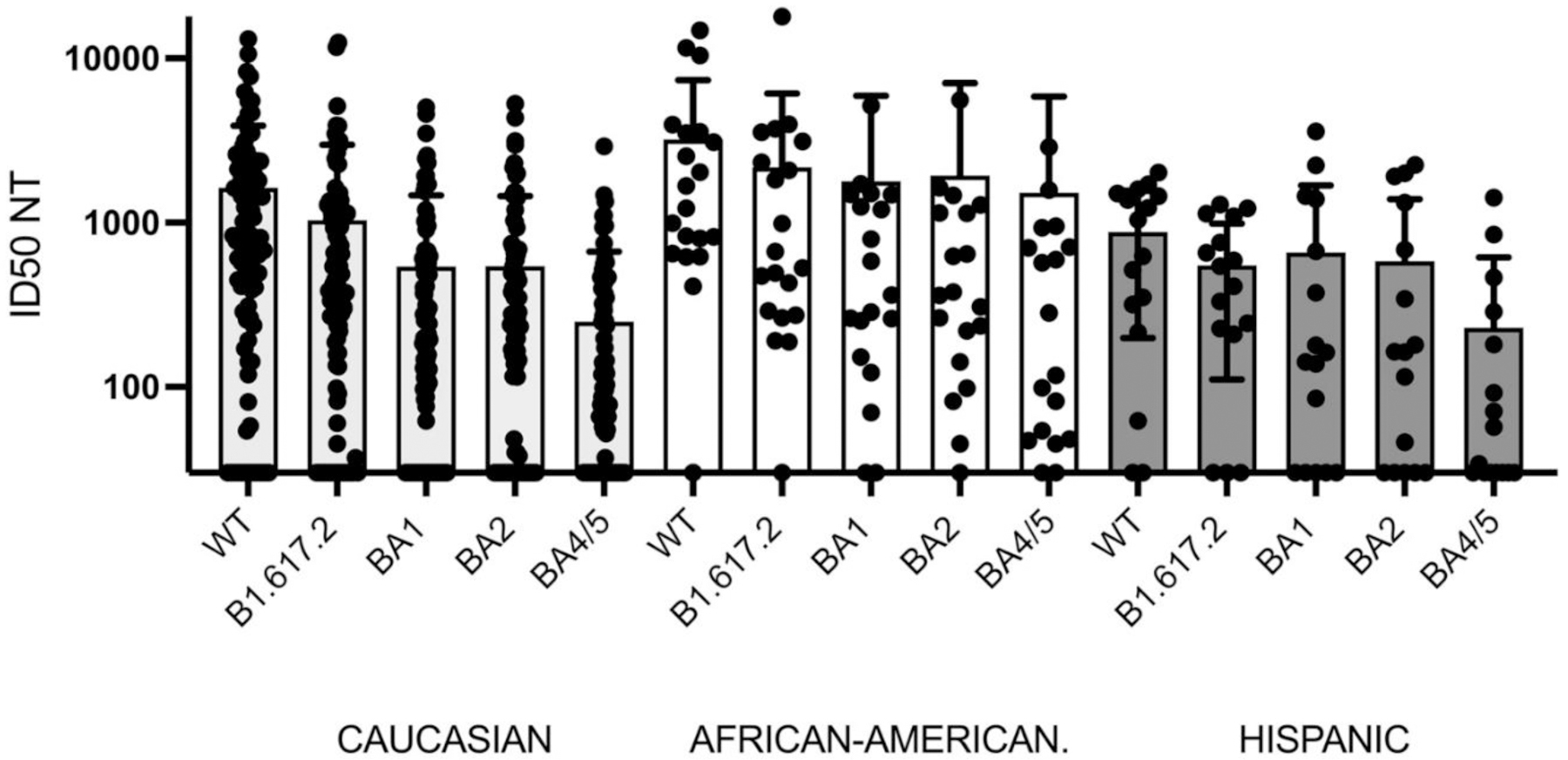
Effect of ethnicity on post-booster WT, B1.617.2, BA1, BA2, and BA4/5 NT. African Americans had significantly greater post NT with F’s of 4.4, 2.7, 3.9,, 3.5, and 4.7 and P’s of 0.01, 0.07, 0.02, 0.03, and .01 for WT, B1.617.2, BA1, BA2, and BA4/5 versus Caucasians or Hispanic by one-way ANOVA. The median post NTs for Caucasian, African Americans and Hispanics were 812, 1,450 and 833 for WT; 385, 598 and 477 for B1.617.2; 177, 473 and 152 for BA1; 214, 370 and 164 for BA2; 250, 1524 and 229 for BA4/5.

**Table 1.** Demographics and Medical Histories*

Property	Full Cohort n = 140	Controls n = 32	Solid Tumor n= 55	Lymphoma n = 19	CLL n = 22	Myeloma n = 12
Age(yr,mean,IQR)	74 (68–79)	74(67–83)	73(66–78)	73(69–77)	75(69–77)	75(69–85)

Gender (M,F)	137M/3F	32M/0F	54M/1F	19M/0F	21M/1F	11M/1F

Ethnicity (C,A,H,N,P,E)	97C, 21A, 18H, 2N, 1P,1E	21C, 3A, 5H, 2N, 1E	39C, 11A, 4H, 1P	11C, 4A, 4H	19C, 1A, 2H	6C, 3A, 3H

COVID (Pre, Post)	14 (8,6)	4 (2,2)	2 (1,1)	1 (1,0)	3 (3,0)	4 (1, 3)
Severity (O,D,U)	4O, 7D, 3U	1O, 2D, 1U	1O, 1D	1D	2O, 1D	2D, 2U

Disease Activity (Tx, No Tx)	79 Tx, 61 noTx	2 Tx, 30 no Tx	39 Tx, 16 no Tx	12 Tx, 7 no Tx	15 Tx, 7 no Tx	11 Tx, 1 no Tx

BoneMarrowTransplant	2	1	0	0	0	1

Anti-CD20 antibody	15	0	0	12	3	0
Recent (≤21 mos) Past (>21 mos)	4 Recent, 11 Past			4 Recent, 8 Past	0 Recent, 3 Past	

* IQR, interquartile range; M, male; F, female; C, Caucasian; A, African American; H, Hispanic; N, native American; P, Pacific Island American; E, Middle Eastern American; O, outpatient; D, inpatient; U, ICU; Tx, treatment; Two veterans received bone marrow transplants including one myeloma patient given two autologous stem cell transplants with the last given ten months before this study and one control with chronic myeloid leukemia who received an allogeneic bone marrow transplant 20 years prior to this study. The three subjects with COVID-19 from the control cohort requiring inpatient or ICU care did not show evidence of immunosuppression—one had stable monoclonal gammopathy for years with an M-spike of 1g/dL and otherwise normal immunoglobulins and his anti-spike antibody level was 20,100AU/mL prior to infection, another control subject had stable mild polycythemia with Hgb in 15g/dL range and anti-spike IgG of 18,460AU/mL prior to infection and the last control had chronic myelomonocytic leukemia not requiring treatment and his anti-spike IgG was 50,000AU/mL on admission.

**Table 2.** Disease Effect on Anti-spike protein IgG levels*

Cohort	Pre-boost IgG (AU/mL ± SD)	Post-boost IgG (AU/mL ± SD)	Ratio	Two-tailed Student’s P
All	5,903 ± 12,530	30,362 ± 19,699	5	<0.0001
Control	8,080 ± 15,537	32,180 ± 19,202	4	<0.0001
Solid Tumor	2,478 ± 5,809	30,350 ± 18,154	12	<0.0001
Myeloma	10.054 ± 17,525	17,347 ± 20,402	1.7	0.36
B cell Lymphoma	3,540 ± 9,333	21,467 ± 19,58*6*	6	0.0009
CLL	6,022 ± 13,171	22,767 ± 23,052	4	0.005
All B malignancy	6,045 ± 13,084	21,074 ± 20,974	3.5	<0.0001
Untreated Solid Tumor	2,906 ± 6,351	33,526 ± 14,508	12	<0.0001
Treated Solid Tumor	2,303 ± 5,650	29,047 ± 19,475	12	<0.0001
Untreated B cell Lymphoma	7,150 ± 15,210	30,913 ± 16,991	4	0.02
Treated B cell Lymphoma	1,434 ± 1,739	15,957 ± 19,502	11	0.02
Untreated CLL	1,305 ± 1,746	19,539 ± 21,313	15	0.04
Treated CLL	8,223 ± 15,576	24,273 ± 24,387	3	0.04
Solid Tumor Untreated Post/Solid Tumor Treated Post			1.2	0.41
Solid Tumor Post/Control Post			1.1	0.76
Untreated CLL Post/Treated CLL Post			0.8	0.66
Untreated B cell Lymphoma Post/Treated B cell Lymphoma Post			1.9	0.11
Control Post/B cell Lymphoma Post			1.5	0.08
Solid Tumor Post/B cell Lymphoma Post			1.4	0.08
B cell Lymphoma Post/CLL Post			0.9	0.84
B cell Lymphoma Post/Myeloma Post			1.2	0.56
Control Post/CLL Post			1.4	0.14
Solid Tumor Post/CLL Post			1.3	0.13
CLL Post/Myeloma Post			1.3	0.5
Control Post/All B malignancy Post			1.5	0.02
Solid Tumor Post/All B malignancy Post			1.4	0.02
All Untreated	6,067 ± 13,336	31,421 ± 18,180	5	<0.0001
All Treated	4,548 ± 10,716	23,719 ± 20,717	5	<0.0001
All Untreated Post/All Treated Post			1.3	0.02
All Anti-CD20 Therapy	4,135 ± 10,495	25,051 ± 21,150	6	0.002
Recent (<21 mos) anti-CD20 Therapy	261 ±448	130 ± 199	0.5	0.61
Past (>21 mos) anti-CD20 Therapy	5,543 ± 12,082	34,113 ± 16,955	6	0.0002
Never anti-CD20 Therapy	4,900 ± 11,416	27,318 ± 19,883	5.6	0.0001
Recent anti-CD20 Post/Past anti-CD20 Therapy Post			262	0.002
Recent anti-CD20 Post/Never anti-CD20 Therapy Post			214	0.006

* Ratio is post anti-S IgG/pre anti-S IgG.

**Table 3.** Disease Effect on Serum Neutralization Titers*

Cohort	WT Pre	WT Post	Ratio	P value	B1.617.2 Pre	B1.617.2 Post	Ratio	P value	BA1 pre	BA1 post	Ratio	P Value	BA2 Pre	BA2 Post	Ratio	P Value
All	749±3438	1874±2858	2.5	.0036	530±3193	1180±2303	2.2	.55	118±544	772±1887	6.5	..0001	105±576	807±2251	7.7	.0004
AllWT/B.1.617.2NT			.61	..03												
All WT/BA1 NT			.41	.0002												
All WT/BA2 NT			.43	.0008												
Controls	477±1559	2089±3019	4	.01	245±925	1252±1423	5	.001	86±180	980±1480	11	.001	123±399	1093±1745	9	.003
Solid Tumor	191±575	1441±1352	7	<.0001	82±215	743±735	9	<.0001	36±32	505±652	14	<.0001	37±34	470±534	13	<.0001
Control/Solid Tumor Post			1.4	.175			1.7	.03			1.9	.04			2.3	.02
Untreated Solid Tumor	201±518	1716±1026	8	<.0001	120±325	748±469	6	.0001	32±5	452±438	14	.0006	31±5	549±591	18	.0015
Treated Solid Tumor	185±602	1329±1461	7	<.0001	87±153	742±851	8	<.0001	38±39	526±726	14	<.0001	39±40	450±516	12	<.0001
Untreated/Treated Solid Tumor Post			1.3	0.32			1	.98			.9	.66			1.2	.35
Myeloma	454±848	1517±3192	3.3	.32	241±433	609±1094	2.5	.3	376±766	293±523	.8	.78	219±430	295±516	1.3	.71
Control/Myeloma Post			1.4	.54			2	.2			3	.1			4	.16
Solid Tumor/Myeloma Post			.9	.99			1.2	.63			1.7	.34			1.6	.32
CLL	1991±5862	2294±3800	1	.84	694±2370	1640±3609	2	.31	294±1229	699±1277	2	.3	255±1041	711±1500	3	.25
Control Post/CLL Post			.8	.9			.8	.6			1.4	.5			1.5	.4
Solid Tumor/CLL Post			.6	.15			.5	.08			.7	.4			.7	.3
Untreated CLL	91±146	815±634	9	.01	30±0	429±560	14	.08	30±0	374±396	12	.04	30±0	231±269	8	.07
Treated CLL	2878±6993	3034±4501	1	.94	1004±2848	2245±4325	1	.4	417±1488	862±1533	2	.4	359±1261	952±1800	2.6	.3
Untreated/Treated CLL Post			.27	.22			.19	.29			.43	.42			.24	.31
B cell Lymphoma	1569±6467	2630±4380	1.7	.57	2300±8142	2227±4372	1	.97	43±55	1627±4539	38	.14	31±6	1762±7198	57	.18
Untreated Lymphoma	4152±10636	4755±5700	1.1	.89	6172±13085	4311±6216	.7	.74	66±91	3076±6908	47	.27	33±9	2711±8556	112	.28
Treated Lymphoma	62±75	1142±2542	18	.15	41±26	767±1577	19	.12	30±0	613±1428	20	.17	30±0	378±972	13	.2
Untreated/Treated Lymphoma Post			67	.09			151	.1			2	.29			1	.24
Control/Lymphoma Post			.8	.6			.6	.08			.6	.45			.6	.8
Solid Tumor/Lymphoma Post			.5	.3			.3	.03			.3	.3			.3	.7
Myeloma/Lymph Post			.6	.5			.3	.08			.2	.4			.2	.4
CLL/Lymphoma Post			.9	.5			.7	.09			.4	.4			.4	.4
All B malignancy	1455±5233	2457±4256	1.7	.3	1112±4966	1689±3520	7	.5	214±845	1003±2837	5	.05	165±680	1108±3520	7	.05
Control/All B Post			.9	.7			.7	.5			1	.9			1	.9
Solid Tumor/All B Post			.6	.1			.4	.05			.5	,2			.4	.2
All Therapy	725±3189	1624±2694	2.2	.06	272±1275	1006±2213	3.7	.01	166±715	570±1003	3.4	.005	130±574	518±956	4	.003
All Untreated	780±3763	2181±3093	2.8	.03	864±4616	1393±2504	1.6	.44	56±121	1019±2582	18	.004	74±288	1161±3171	16	.009
Untreated/Treat Post			1.3	.26			1.4	.33			1.8	.17			2.2	.1
Recent anti-CD20	32±4	76±92	15	.37	30±0	105±150	3.5	.37	30±0	30±0	1	1	30±0	30±0	1	1
No anti-CD20	604±2659	1703±2560	2.8	.0009	246±1119	1020±1810	4.1	.0001	127±576	638±1026	5	.0001	115±498	636±1107	5.5	.0001
No anti-CD20/Recent Anti-CD20			25	.2			12	.35			26	.42			28	.49

* The small number (n = 4) for recently anti-CD20 antibody treated subjects likely impacted the statistical significance.

**Table 4.** Disease Effect on Serum Omicron BA4/5 Neutralization Titers*

Cohort	BA4/5 pre	BA4/5 post	Ratio	P value
All	72 ± 250	443 ± 1710	6.1	.01
All WT/BA4/5 post			4.2	.0001
Control	117 ± 398	517 ± 735	4.4	.01
Solid Tumor	33 ± 24	243 ± 298	7.4	.0001
Control/Solid tumor post			2.1	.02
Untreated Solid Tumor	30 ± 0	255 ± 272	8.5	.003
Treated Solid Tumor	35 ± 28	237 ± 311	6.8	.0002
Untreated/Treated Solid Tumor post			1.1	.85
Myeloma	134 ± 268	231 ± 479	1.7	.56
Control/Myeloma post			2.2	.26
Solid Tumor/Myeloma post			1.1	.92
CLL	108 ± 368	308 ± 658	2.9	,22
Control/CLL post			1.7	.30
Solid Tumor/CLL post			0.8	.55
Untreated CLL	30 ± 0	116 ± 117	3.9	.08
Treated CLL	145 ± 446	405 ± 793	2.8	.28
Untreated/Treated CLL post			.29	.36
Lymphoma	30 ± 0	1290 ± 4623	43	.24
Untreated Lymphoma	30 ± .3	2933 ± 7178	98	.31
Treated Lymphoma	30 ± 0	139 ± 289	4.6	.20
Untreated/Treated Lymphoma post			21	.23
Control/Lymphoma post			.4	.37
Solid Tumor/Lymphoma post			.19	.1
Myeloma/Lymphoma post			.18	.48
All B malignancies	86 ± 268	640 ± 2783	7.4	.15
Control/All B malignancies post			.8	.81
Solid Tumor/All B malignancies post			.38	.30
All Therapy	75 ± 224	254 ± 456	3.4	.002
All Untreated	68 ± 282	677 ± 2497	10	.06
Untreated/Treated post			2.7	.15
Recent anti-CD20 antibody	30 ± 0	30 ± 0	1	1
No anti-CD20 antibody	77 ± 264	299 ± 474	3.9	.0001
No anti-CD20 antibody/Recent anti-CD20 antibody post			10	.26

* The small number (n = 4) for recently anti-CD20 antibody treated subjects likely impacted the statistical significance.
