# Supplementary material for: The Effects of an mRNA Covid-19 Vaccine Booster on Immune Responses in Cancer-Bearing Veterans
Source: Med Res Arch. Author manuscript; Available in PMC 2022 Nov 17. (PMC9670257; doi:10.18103/mra.v10i7.2932)
Supplement: supplementary material [file NIHMS1829772-supplement-supplementary_material.pdf]

All supplementary material is included within the single manuscript file. There are no other files.
